# Sample preparation for proteomic analysis using a GeLC-MS/MS strategy

**DOI:** 10.14440/jbm.2016.106

**Published:** 2016-07-12

**Authors:** Joao A. Paulo

**Affiliations:** Department of Cell Biology, Harvard Medical School, Boston, MA 02115, USA

**Keywords:** biomarkers, GeLC-MS/MS, mass spectrometry, proteomics

## Abstract

In-gel digestion coupled with mass spectrometric analysis (GeLC-MS/MS) is a cornerstone for protein identification and characterization. Here I review this versatile approach which combines classical and modern biochemistry strategies and allows for targeted and proteome-wide analyses. Starting with any protein sample, reduced and alkylated proteins are precipitated prior to fractionation by SDS-PAGE. Proteins are in-gel digested and the resulting peptides are extracted and desalted for downstream LC-MS/MS analysis. GeLC-MS/MS leverages the advantages of both traditional SDS-PAGE visualization and protein fractionation with the robust protein and post-translational modification identification and quantitation capabilities of state-of-the-art mass spectrometry-based technology. As such, this strategy allows for the visible assessment of protein amount and quality, prior to analysis via virtually any mass spectrometry platform. Moreover, gel extracted peptides may be derived from any sample type—*e.g.*, from cell culture, tissue, body fluid, or recombinantly-expressed protein—and are fully compatible with isobaric tagging. GeLC-MS/MS is an invaluable technique for proteomic analyses.

## BACKGROUND

In-gel digestion coupled with mass spectrometric analysis is a powerful, yet relatively uncomplicated strategy to identify and characterize proteins. Pre-fractionation of protein mixtures, before and/or following enzymatic digestion, is typically required prior to mass spectrometric analysis. Separating proteins into several fractions can improve analytical depth by decreasing sample complexity. In PAGE-based fractionation strategies, the polyacrylamide gel matrix is an excellent sieve to separate proteins from low molecule weight compounds and buffer components, which can interfere with downstream mass spectrometric analyses. The origins of in-gel digestions may be traced back to the early 1990s. This strategy has since been attributed to Mann and colleagues [1], although prior to being applied to mass spectrometry, in-gel-protein digestion was well-established in Edman degradation analysis [2-4].

In-gel digestion offers several benefits over in-solution digestions. Foremost, this strategy can target and identify specific gel bands or spots of a particular molecular weight and/or isoelectric point and is useful for investigating proteins which differ under certain cellular conditions. As the procedure allows for protein visualization, colorimetric estimates of protein concentration can be verified prior to protein digestion. Furthermore, for samples which are prone to degradation, assessing sample quality is imperative and can be determined visually following SDS-PAGE. For example, in-gel tryptic digestions have been vital to mass spectrometry-based proteomic analysis of pancreatic fluid, and of other fluids of the digestive tract [5-10].

**Figure 1.**
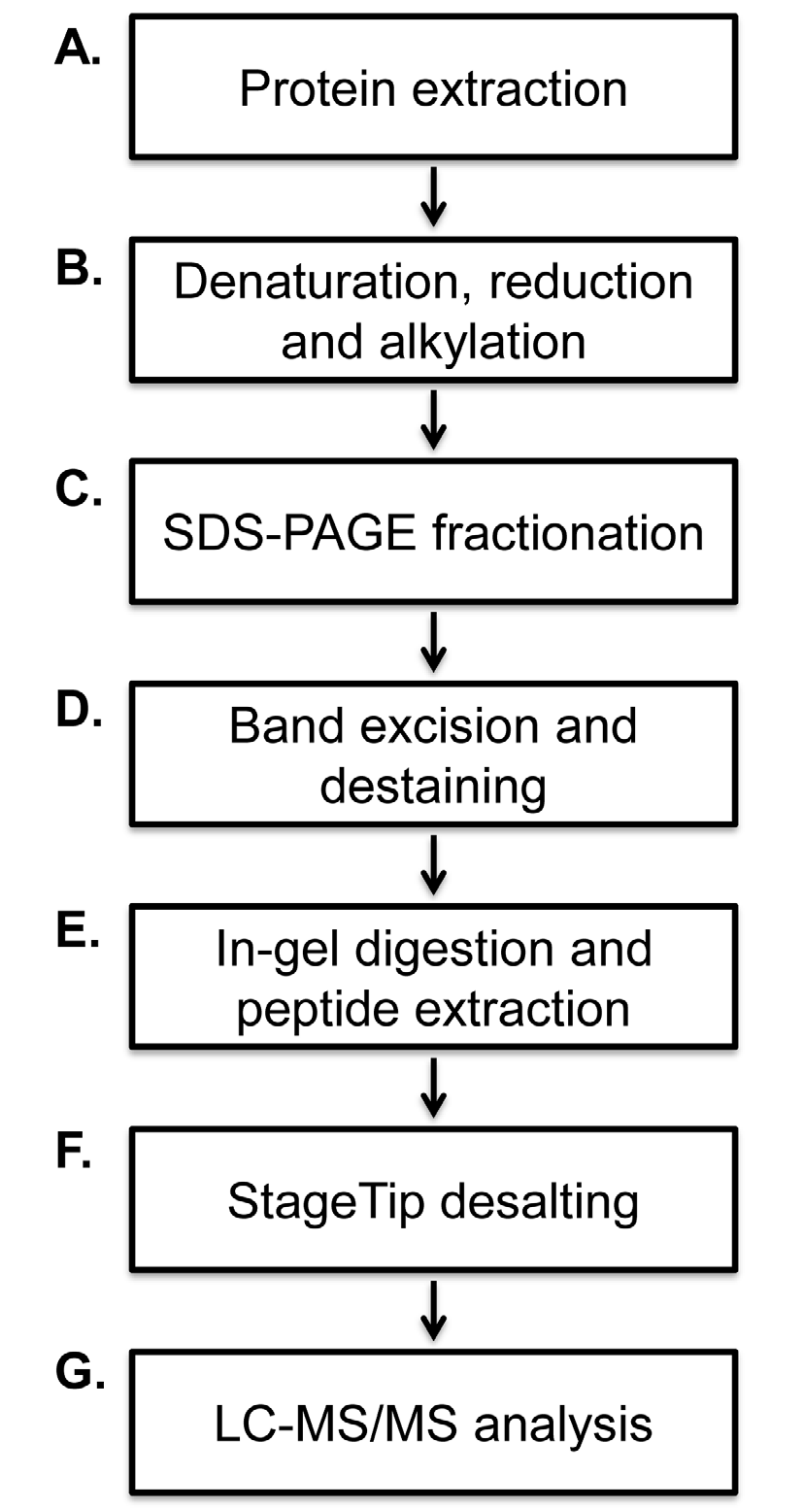
**General workflow of a GeLC-MS/MS experiment. A.** Protein extraction, **B.** SDS-PAGE fractionation, **C.** Band excision and destaining, **D.** In-Gel reduction and alkylation (optional), **E.** In-gel enzymatic digestion, **F.** Clean-up prior to mass spectrometry analysis via StageTips (optional, but highly recommended), and **G.** Final preparation of samples for mass spectrometry.

As outlined in **Figure 1**, the procedure encompasses: A. Protein extraction from complex biological matrix typically via protein fractionation techniques, B. Reduction and alkylation prior to SDS-PAGE, C. SDS-PAGE fractionation, D. Band excision and destaining, E. In-gel enzymatic digestion, F. Desalting prior to mass spectrometry analysis via StageTip, and G. Analysis via liquid chromatography coupled to mass spectrometry. Although the protocol focuses on processing gel slices from Coomassie-stained, one dimensional SDS-PAGE, the general procedure is applicable to multi-dimensional gel electrophoresis, in addition to a variety of mass spectrometry-compatible protein staining procedures.

## MATERIALS

All solutions should be prepared with MilliQ® water or the equivalent, and HPLC quality solvents should be used. Unless specified otherwise, all reagents should be prepared and stored at room temperature. In addition, local waste disposal guidelines and regulations should be followed.

### Reagents

• Acetic acid (Sigma-Aldrich, cat. # A6283)
• Acetonitrile (BDH, cat. # BDH20864.400)
• DTT, dithiothreitol, Cleland's reagent (Sigma-Aldrich, cat. # D0632)
• EPPS, 3-[4-(2-hydroxyethyl)-1-piperazinyl]-1-propanesulfonic acid (Sigma-Aldrich, cat. # E9502)
• Formic acid (EMD, cat. # 1.11670.1000)
• IAA, iodoacetamide (Sigma-Aldrich, cat. # A3221)
• Methanol (BDH, cat. # BDH83639.400)
• NuPAGE 4X SDS Sample Buffer (LifeTechnologies, cat. # NP0008) or equivalent.
• NuPAGE Bis-Tris precast gradient gels (LifeTechnologies, cat. # NP0324) or equivalent.
• SimplyBlue Coomassie Staining (LifeTechnologies, cat. # LC6060) or equivalent.
• TCA, trichloroacetic acid (Sigma-Aldrich, cat. # T6399)
• TCEP, tris(2-carboxyethyl)phosphine (Pierce, cat. # 20490)
• Trypsin, sequencing grade (Promega, cat. # V5111).

### Solutions

• Destaining Buffer: 50% acetonitrile, 50% 100 mM EPPS pH 8.5 in ultrapure water.
• Digestion Buffer: 100 mM EPPS pH 8.5 in ultrapure water.
• Trypsin Stock: Prepare Trypsin Stock solution by hydrating the lyophilized trypsin (20 µg) with 20 µl of the supplied trypsin storage solution, or 0.1% acetic acid. Aliquot the reconstituted trypsin (5 µl) into separate tubes to minimize freeze-thaw cycles and to increase storage stability. Store unused aliquots at −80°C.
• Trypsin Working Solution: Thaw a Trypsin Stock aliquot on ice. Dilute the stock 10-fold by adding 45 µl ultrapure water.
• Peptide Extraction Solution: 1% formic acid, 75% acetonitrile in ultrapure water.
• DTT stock solution: 500 mM in ultrapure water.
• IAA stock solution: 500 mM in ultrapure water.
• TCEP stock solution: 500 mM in ultrapure water.
• StageTip reconstitution/equilibration buffer: 1% formic acid in ultrapure water.
• StageTip wash buffer: 1% formic acid, 5% acetonitrile in ultrapure water.
• StageTip elution buffer: 1% formic acid, 70% acetonitrile in ultrapure water.
• Mass spectrometry loading buffer: 5% formic acid, 5% acetonitrile.
• Empore C18 Membrane Disk (3M, cat. # 2215)
• Monoject 19 gauge × 1.5 inch long blunt needle (Kendall, cat. # SWD202389)
• 200 µl pipette tips with narrow bores and no filters (e.g., Mettler-Toledo, cat. # GP-L250S)

## PROCEDURE

The in-gel digestion protocol described herein can be applied to both cell-based and secreted proteins (including bodily fluids) [5-10]. Cells can be lysed via a variety of mechanical methods (*e.g.*, needle lysis, Dounce homogenization, sonication) and/or buffer systems (*e.g.*, 8 M urea, 2% SDS). Likewise, secreted proteins can be denatured in a similar manner. Moreover, protease inhibitor cocktails can be added to prevent undesirable non-specific proteolysis of extracted proteins. In addition, protein concentration should be determined (*e.g.*, with Bradford assay, bicinchoninic acid (BCA) assay or equivalent.

### Protein Denaturation, Reduction and Alkylation

For GeLC-MS/MS analysis, samples must be reduced and alkylated to enhance protein digestion by cleaving and preventing the re-formation of disulfide bonds. Reduction and alkylation can be performed either before fractionating the protein on the gel, or after staining and destaining of the gel. I recommend performing reduction and alkylation prior to GeLC-MS/MS, as it typically results in a clearer protein profile. Greater sequence coverage may be gained by reduction and alkylation, which is particularly useful if a protein or specific peptide of interest in a targeted experiment had abundant cysteines. Upon alkylation, iodoacetamide (IAA) reacts with the cysteine to form carboxyamidomethylcysteine, which results in an increase in mass of 57.021 Da. IAA may not be the ideal alkylation reagent for all studies. For example, IAA can modify a fraction of lysine residues twice resulting in a mass difference of 114.043 Da, similar to that of a GG tag generated by the tryptic digestion of ubiquitin [11]. Similarly, alkylation with chloroacetamide may also produce this artifact at high temperature [12], as such N-ethylmaleimide (NEM) is typically used as the alkylating reagent for ubiquitination studies.

1. Add 5 mM of TCEP to the tube containing the sample and incubate at room temperature for 20 min to reduce disulfide bonds.
2. Add 10 mM of IAA to alkylate free cysteines. Incubate sample in the dark at room temperature for 20 min.
3. Add 10 mM of DTT to the tube to quench the IAA. Incubate sample in the dark at room temperature for 20 min.

### Protein extraction

Regardless of the sample origin (*i.e.*, cell culture, body fluid, immunoprecipitate), proteins must be extracted from non-proteinaceous matter to produce sharp, clear, and distinct bands indicative of proper fractionation. Many precipitation methods have been established to extract proteins from various sample types. A screening of available methods may be warranted to maximize protein yield for a particular sample. Below are outlined two methods for general protein precipitation which can remove compounds that are detrimental to the quality of protein analysis via SDS-PAGE. First, methanol chloroform precipitation which is useful for samples >500 µg/ml [13]; and second, trichloroacetic acid (TCA)-acetone precipitation which is useful for more dilute samples and larger volumes.

### Methanol-chloroform precipitation (all steps are performed at room temperature)

4. Dilute sample so that its volume is ~100 µl in a 1.5 ml microcentrifuge tube (volumes can be scaled up, as necessary).
5. Add 400 µl of 100% methanol and vortex for 5 s.
6. Add 100 µl of 100% chloroform and vortex for 5 s.
7. Add 300 µl of water and vortex for 5 s.
8. Centrifuge for 1 min at 14,000 g.
9. Remove aqueous layer (top) and organic layer (bottom), but retain the middle protein disk.
10. Add 400 µl of 100% methanol and vortex for 5 s.
11. Centrifuge for 2 min at 14,000 g.
12. Remove as much methanol as possible without disturbing the pellet.
13. Air dry pellet.

### TCA-Acetone precipitation (alternative to methanol-chloroform precipitation)

14. Add 12.5% sample volume of ice-cold 100% TCA to the sample.
15. Vortex briefly (5 s) and incubate at 4°C for 1 h.
16. Centrifuge at 10,000 g at 4°C for 15 min.
17. Decant supernatant and resuspend pellet in 1 ml of cold (-20°C) 100% acetone.
18. Incubate at −20°C for 30 min.
19. Centrifuge at 10,000 g at 4°C for 15 min.
20. Decant supernatant and resuspend pellet in 1 ml of cold 100% acetone.
21. Centrifuge at 10,000 g at 4°C for 15 min.
22. Repeat steps 20 and 21.
23. Air dry pellet.

### SDS-PAGE fractionation

Proteins may be fractionated to varying degrees depending on sample complexity. For example, when dealing with relatively small proteomes, purified proteins, or targeted assays, time of separation may be shorter and fewer gel slices (or even a single slice [14]) will be sufficient. However, more complex samples, such as whole cell lysates, may require 12–24 fractions in attempts to maximize proteome coverage.

24. Dissolve pellet in 50 μl of 1 × SDS Sample Buffer.
25. Load sample (20–100 μg) on the SDS-PAGE gel (*i.e.*, a 4–12% gradient NuPAGE gel or the equivalent).
26. Proteins can then be fractionated by SDS-PAGE under any buffer system (*e.g.*, at 150 volts in MES (2-(N -morpholino)ethanesulfonic acid) buffer for 45 min).
27. Following fractionation, rinse gel in deionized water for 10 min.
28. Stain the gel with SimplyBlue Coomassie, or equivalent stain, for 1 h. Detection limits of various common stains are listed in **Table 1**.
29. Destain overnight in deionized water.

### Band excision and destaining

**Figure 2.**
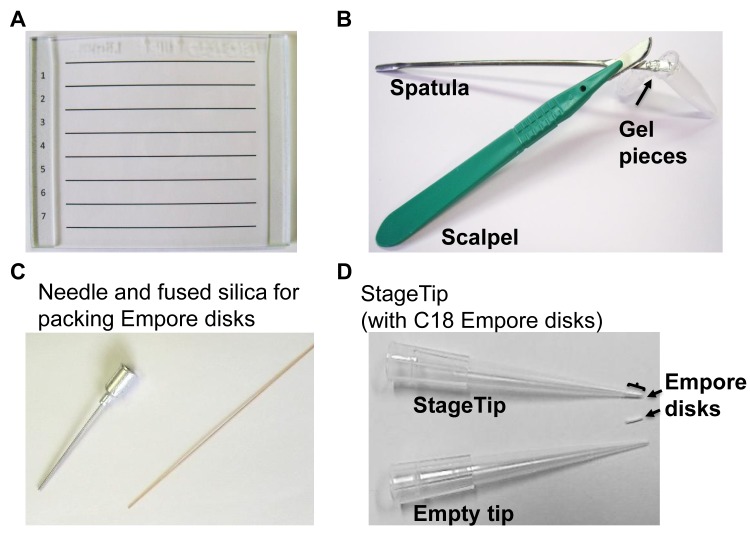
**Some necessary instruments for GeLC-MS/MS.** For excision of gel regions of interest: **A.** glass cutting plate and template, **B.** as scalpel and spatula are used to cut gel regions for transfer into microcentrifuge tubes, and **C.** a blunt-ended needle and fused silica are used to pack the Empore disk into a pipette tip to construct **D.** StageTips.

Ensure that this procedure is performed in an environment that is as “keratin-free” as possible. All scalpels, spatulas, razor blades and surfaces should be properly cleaned immediately prior to use. A lint-free wipe soaked in TFA can be used to clean the working surfaces. For gel band excision, we recommend cutting the gel on a glass plate or other clear, clean surface, as this will allow for the placement of a preprinted template, which is particularly useful for excising equally spaced gel slices (**Fig. 2A**). Other strategies may cut bands according to protein density, rather than using gel slices of equal proportions. Such will be ideal for immunoprecipitations so as to avoid dense antibody bands and highly abundant proteins. Alternatively, the whole gel can be destained prior to gel cutting.

This gel band excision and destaining procedure is designed for Coomassie, or mass spectrometry-compatible fluorescent dye-stained polyacrylamide gels. Alternative destaining procedures may be required for silver- or zinc-stained protein bands[15]. For silver staining, it is imperative that the treatment with the cross-linking reagent (*i.e.*, glutaldehyde) is omitted during the staining procedure [16].

30. Use a scalpel (or clean razor blade) to excise protein sections of interest - typically 10–25 sections for a mini-gel (**Fig. 2B**). Note that excising gel slices with a scalpel and cutting on a small spatula may facilitate the process of transferring the gel pieces to the microfuge tube for subsequent analyses.
31. Cut the gel section into 1 × 1 to 2 × 2 mm pieces.
32. Place pieces into a 1.5 ml low-binding microcentrifuge tube.
33. Add 200 µl of Destaining Buffer to gel pieces.
34. Incubate sample at 37°C for 30 min with shaking.
35. Remove and discard Destaining Buffer from the tube.
36. Repeat steps 33 to 35 twice or more until the Coomassie dye is no longer visible in the gel.

### In-gel enzymatic digestion

Trypsin is used often for in-gel digestions as it is highly specific, relatively inexpensive, and by cleaving at arginine and lysine amino acid residues, ensures that a positive charge is present at the C-terminus, in addition to the N-terminus, thereby resulting in doubly charged peptides. Moreover, trypsin is active in a wide range of basic buffers and is inactivated by the addition of acid. However, different applications may require an enzyme other than trypsin. In lieu of trypsin, other enzymes, such as chrymotrypsin, Lys-C, Glu-C, and Asp-N among others, have been used successfully for in-gel digestions.

37. Dehydrate gel pieces by adding 500 µl of acetonitrile.
38. Incubate for 10 min at room temperature.
39. Carefully remove acetonitrile and allow gel pieces to air-dry for 10–15 min at room temperature.
40. Swell gel pieces by adding 10 µl (200 ng) of trypsin solution to the microcentrifuge tube. Note that the recommended amount of trypsin per digest is 200 ng when using a 7 cm mini-gel that is divided into 7–10 slices. However, if protein band/gel section contains less than ~20 ng of protein, trypsin may be diluted accordingly.
41. Incubate on ice for 20 min.
42. Add 50 µl Digestion Buffer to the tube. Incubate sample at 37°C overnight with shaking. Ensure that the gel pieces are covered with sufficient buffer to account for evaporation so that the gels do not dry overnight.
43. Remove digestion mixture and place in a clean tube. To extract peptides, add 50 µl of 1% formic acid solution to the gel pieces and incubate for 15 min. This acidification step also serves to inactivate trypsin, thus arresting additional enzymatic activity.
44. Remove extraction solution and add to digestion mixture (step 43).
45. To extract more hydrophobic peptides, add 50 µl of 1% formic acid, 75% acetonitrile solution to the gel pieces and incubate for 15 min.
46. Remove extraction solution and add to digestion mixture (step 43).
47. Vacuum centrifuge the sample to dryness.
48. Samples may now be purified further using StageTips.

### Clean-up prior to mass spectrometry analysis via StageTips (optional, but highly recommended)

The StageTip procedure described below has been developed with input from several previous publications [17,18]. StageTip sample clean-up is often omitted due to concern of peptide loss or the additional time and effort required. However, performing this additional procedure prevents small polyacrylamide gel particles from entering and potentially damaging the liquid chromatography system, in addition to improving spectral quality by eliminating many interfering ions.

### Constructing StageTips

49. Using a blunt needle, excise 4–6 disks (often referred to as “cookies”) from the Empore membrane (**Fig. 2C**).
50. Insert a short piece of solid fused silica or thin metal rod into the blunt needle to be used to gently deposit these disks into the pipette tip. The disks should not protrude from the opening to the tapered tip (**Fig. 2D**).
51. Repeat as necessary, but for in-gel digestions, 4–6 disks should suffice.

### Performing StageTip clean-up

The StageTip procedure can be performed manually using a single or multichannel pipette, or using a tabletop centrifuge. Typically, centrifugation for 2 min at 1500 g (~4000 RPM) is sufficient to pass the volumes listed below. However, variations in tips, centrifuges, and number of disks used can affect the time and speed necessary for proper centrifugation. As such, “2 min at 1500 g” should be used as a guide from which to optimize further this procedure.

52. Reconstitute dried samples in 100 µl of 1% formic acid (FA)/5% acetonitrile (ACN).
53. Wet StageTip with 100 µl 100% methanol.
54. Centrifuge for 2 min at 1500 g.
55. Repeat Steps 53 and 54.
56. Pre-wash StageTip with 50 µl of 1% FA, 70% ACN.
57. Centrifuge for 2 min at 1500 g.
58. Repeat Steps 56 and 57.
59. Equilibrate StageTip with 50 µl of 1% FA, 5% ACN.
60. Centrifuge for 2 min at 1500 g.
61. Repeat Steps 59 and 60.
62. Load sample onto column.
63. Centrifuge at half speed for twice as long (*e.g.*, at 750 g for 4 min).
64. Wash off non-peptide moieties with 50 µl of 1% FA, 5% ACN.
65. Centrifuge for 2 min at 1500 g.
66. Repeat Steps 64 and 65.
67. Elute peptides with 25 µl of 1% FA, 70% ACN.
68. Centrifuge for 3 min at 1500 g.
69. Repeat Steps 37 and 68.
70. Speed-Vac the sample to dryness.

### Final preparation of samples for mass spectrometry

71. Reconstitute the samples in 20 µl of 5% FA, 5% ACN. The acetonitrile in the sample buffer aides in removing many of the singly charged species from entering the mass spectrometer. Note that some very hydrophilic peptides (particularly phosphopeptides) may elute at 5% ACN and so the acetonitrile may be omitted or decreased to 1-5-3% in the wash buffer.
72. The sample may now be analyzed by LC-MS/MS.

## ANTICIPATED RESULTS

The data quality produced in any mass spectrometry experiment is directly dependent on the quality of the sample being analyzed. Following analysis via mass spectrometry [19, 20], data may be processed by one or several search. The number of proteins identified via GeLC-MS/MS may range from under 100 to several thousand, contingent on the level of protein complexity of the gel slice being analyzed. These proteins may be subjected to gene ontology analysis and interrogated for post translational modifications. Analysis of single bands representing purified proteins can uncover post-translational modifications, or interacting proteins from immunoprecipitations. For immunoprecipitations, gel fractionation offers a method to potentially avoid antibody light and heavy chains while also readily eliminating small molecules - such as Triton X-100 - that can interfere with mass spectrometric analysis.

**Table 1:** Detection limits for most common protein stains.

Stain	Detection limit (ng per gel band)	References
Coomassie	30–100	[24, 25]
Colloidal Coomassie	1–16	[24, 26]
Silver stain	0.5–1	[27, 28]
DiGE (Cy2/Cy3/Cy5)	0.025	[29]

**Figure 3.**
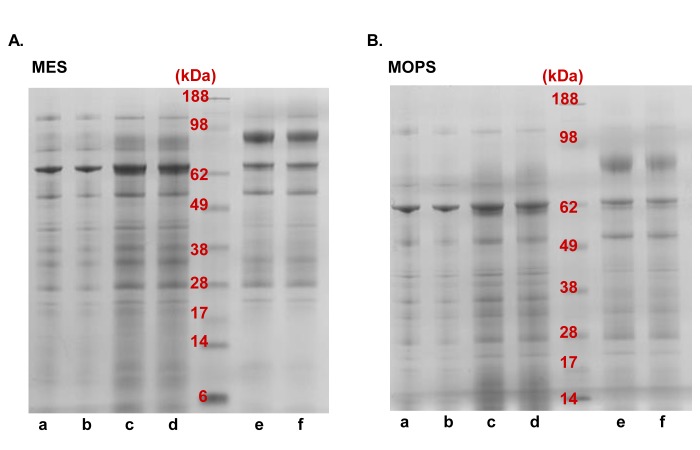
**Gel running buffers result in different protein patterns. A** and **B.** Equal amounts of protein samples a-f were fractionated using both (A) MES and (B) MOPS SDS-PAGE running buffers. Differences in the protein profiles are apparent.

Although the GeLC-MS/MS strategy is universally applicable to protein identification, several caveats persist. For example, artifactual modifications may be present in gel-based proteomic analyses. Polymerization of gels approaches only 90%; as such, estimates indicate the presence of 30 mM of free acrylamide in the gel matrix. Thus, unwanted modifications may result if proteins are not reduced and alkylated prior to electrophoresis. Unpolymerized acrylamide can react with cysteine residues, and as such, alkylation may be performed with 1% acrylamide prior to loading proteins onto the gel [21]. In addition, methionine residues are also susceptible to oxidation, potentially resulting from the presence of persulfate in the gel [22]. These modifications are generally accounted for in database searching procedures.

Sample loss in GeLC-MS/MS experiments is common, but careful sample handling may avoid this caveat. GeLC-MS/MS identifications can be reduced by numerous mechanisms including incomplete protein solubilization prior to electrophoresis and imperfect extraction of the peptides following digestion. In GeLC-MS/MS experiments, peptide recovery has been estimated as 70–90% relative to in-solution digestions [23]. Peptide loss also results from absorption to surfaces of pipette tips and microfuge tubes, drying of samples in a vacuum concentrator, and during ionization. Some losses in peptide and protein identifications can be recovered by repeated and/or complementary analysis via orthogonal methodologies, such as in-solution digestions or filter-aided digestion strategies.

Seemingly trivial, care should be taken as to the buffers and gels system used for protein fractionation. For example, either MES (2-(N-morpholino)ethanesulfonic acid) or MOPS (3-(N-morpholino)propanesulfonic acid) running buffer is recommended as the mobile phase for NuPage gels from Life Technologies. However, substantially different protein banding patterns will be produced, depending on which buffer is used (**Fig. 3**). In addition, we recommend using precast gels to improve reproducibility, as these gels show little variation among batches compared to hand-cast gels. Furthermore, gradient gels provide superior separation over a wide molecular weight range and may be beneficial to certain applications.

The GeLC-MS/MS sample preparation protocol, as described herein, is a time-tested methodology that has been used for decades for protein identification. This technique is robust in its ability to remove mass spectrometry incompatible detergents from immunoprecipitated samples and can be used to eliminate non-proteinaceous compounds from body fluids, such as urine, pancreatic and gastroduodenal fluids, as well as fresh tumor tissue and FFPE embedded tissue material. GeLC-MS/MS can be combined with relative quantification approaches, such as isobaric (*e.g.*, iTRAQ or TMT) or SILAC labeling, and label-free quantification, allowing for relative quantitation across sample. As such, this technology remains a cornerstone of mass spectrometry-based proteomics analysis.

## TROUBLESHOOTING

Potential problems and troubleshooting solutions are listed in **Table 2**.

**Table 2.** Troubleshooting table.

Problem	Possible solution
Stained protein on gel displays edge effects within gel lanes or band smearing.	This could be a result of several problems. Adding 1 × sample buffer to any unused wells may often solve this problem. The problem may also stem from additives in the sample buffer, so one should minimize or decrease salts, detergents, and solvents during sample preparation and in sample buffers.
Sample is loaded onto gel, but sample floats out of the well.	Add 10% glycerol to make sample denser than the surrounding buffer.
Gel does not destain quickly prior to cutting.	The addition of one or two crumpled KimWipe tissue will bind residual Coomassie dye, and accelerate the destaining process.
Gel slice remains blue following several washes.	Note that more frequent changes of Destaining Buffer may be necessary to sufficiently destain intensely-stained gel pieces. If gel pieces become dehydrated (white and rigid), destaining becomes less efficient. Alternating Destaining Buffer with a five-minute wash in organic solvent-free Digestion Buffer (100 mM EPPS pH 8.5) may aid the destaining process.
Following digestion, gel slices are dry.	Ensure that the gel pieces are covered with sufficient buffer to account for evaporation so that the gels do not dry overnight.
